# Vierjähriges Mädchen mit traumatischer Karotisdissektion und nachfolgendem Mediainfarkt

**DOI:** 10.1007/s00101-025-01634-2

**Published:** 2026-01-09

**Authors:** Thorsten Walter, Mareike Schimmel, Stefan Siegert, Lars Behrens

**Affiliations:** 1 Klinik für Anästhesiologie und Intensivmedizin, Universitätsklinikum Augsburg, Stenglinstr. 2, 86156 Augsburg, Deutschland; 2 Klinik für Kinder- und Jugendmedizin – Sektion Kinderneurologie, Universitätsklinikum Augsburg, Augsburg, Deutschland; 3 Klinik für Kinderchirurgie, Universitätsklinikum Augsburg, Augsburg, Deutschland; 4 Klinik für Radiologie und Neuroradiologie, Universitätsklinikum Augsburg, Augsburg, Deutschland

Ziel dieses Fallberichtes soll u. a. sein, auf diese insgesamt seltene, jedoch auch im Kindes- und Jugendalter nicht unbedeutende Differenzialdiagnose aufmerksam zu machen.

## Präklinik/Notaufnahme

Unserer Universitätskinderklinik wurde eine vierjährige Patientin (105 cm/20 kg) aus einer peripheren Klinik der unfallchirurgischen und pädiatrischen Grundversorgung zuverlegt. Das Kind hatte am Vormittag des Aufnahmetages einen Rodelunfall erlitten, wobei die Patientin laut einheitlicher Aussage Umstehender mit niedrigem Tempo auf den vor ihr stehenden Schlitten ihres Bruders aufgefahren war; ein Kopfanprall und Bewusstlosigkeit wurden nicht beobachtet. Aufgrund einer Fallneigung nach rechts und Minderung der Armkraft rechts erfolgte die Vorstellung in der Kreisklinik via RTW, durch diese erfolgte – zunächst bei Verdacht auf zervikale Plexusschädigung – die luftgebundene Verlegung in unser Universitätsklinikum.

Die Aufnahme der Patientin erfolgte über das multidisziplinär besetzte Schockraumteam. Mittlerweile erschien die neurologische Symptomatik deutlich rückläufig; der rechte Arm konnte aktiv gebeugt und gestreckt werden. Allerdings war eine verminderte Sprachproduktion bei erhaltenem Sprachverständnis festzustellen. Aufgrund der fluktuierenden Symptome nach mutmaßlichem Dezelerationstrauma wurde im Konsens die Indikation zur nativen Computertomographie des Schädels gestellt, welche ohne Blutungs- oder Frakturnachweis blieb. Nebenbefundlich fand sich eine Aplasie des linken M. sternocleidomastoideus mit Hypertrophie der Gegenseite. Dennoch wurde der Kopf leicht zur rechten Seite gedreht; der Blick war nach links gewendet. Eine passive Kopfdrehung zur linken Seite wurde von der Patientin als schmerzhaft beschrieben. Anamnestische Auffälligkeiten der Halsregion (insbesondere Trauma in der Vorgeschichte) lagen nicht vor.

## Klinischer Verlauf

Unter der Verdachtsdiagnose eines milden Schädel-Hirn-Traumas mit – möglicherweise – zusätzlicher HWS-Distorsion wurde die Patientin gemeinsam mit ihrer Mutter gemäß Leitlinie zur weiteren neurologischen Überwachung auf die kinderchirurgische Normalstation übernommen. Während der ersten Stunden der Überwachung blieb die Schwäche der rechten Körperhälfte weiterhin gering ausgeprägt. Eine verminderte Sprachproduktion wurde der Malcompliance und einer gewissen Müdigkeit der Patientin zugeschrieben.

Circa 4,5 h nach Aufnahme in unserer Klinik zeigte die Patientin eine neurologische Verschlechterung mit Erbrechen und zunehmender Fallneigung zur rechten Seite mit nun schlaffer Parese des rechten Arms. Zudem wurde eine zentrale Fazialisparese rechts festgestellt. Es erfolgte umgehend eine MR-Angiographie in Intubationsnarkose, hier zeigte sich eine mutmaßlich traumatisch bedingte Dissektion der linken A. carotis im Bereich der Pars petrosa mit deutlich vermindertem Flusssignal der zentralen A. cerebri media links mit beginnender Demarkierung im Perfusionsgebiet im Sinne einer akuten Minderperfusion.

## Radiologische Diagnostik und Intervention

**Abb. 1:**
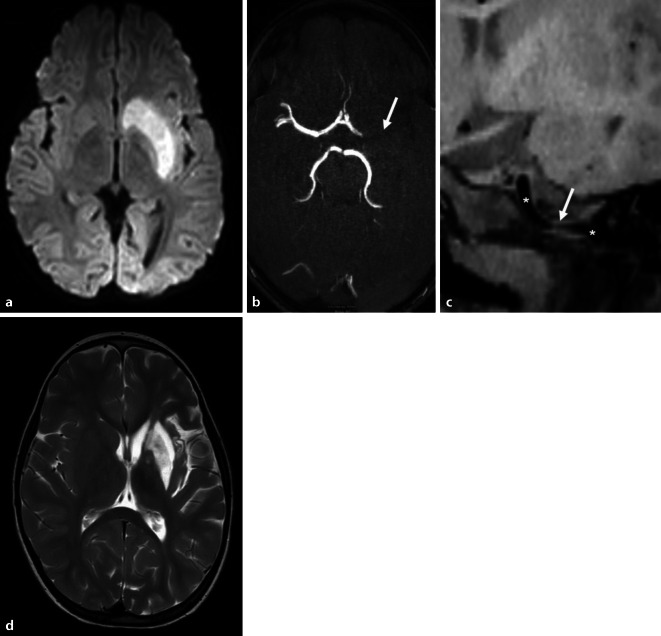
Initiales MRT: **a** Diffusionsgewichtete Sequenz (DWI b1000) mit Infarkt in den Stammganglien links, **b** MR-Angiografie (ToF) mit Verschluss der linken A. cerebri media (*Pfeil*), **c** Native T1-black-blood-Sequenz, schräg-koronar rekonstruiert, mit petröser ACI-Dissektion und Lumeneinengung (*Pfeil*) bei normalem ACI-Kaliber proximal und distal davon (*Sternchen*); **d** Verlaufskontrolle. T2-Sequenz mit Infarktdefekt in den Stammganglien links

Im MRT zeigten sich links ein Stammganglieninfarkt bei Verschluss der terminalen A. carotis interna und des Hauptstamms der A. cerebri media, außerdem ein Wandhämatom im petrösen Abschnitt der A. carotis interna mit Einengung des Gefäßlumens (Abb. 1a–c). Es wurde die Diagnose einer ACI-Dissektion mit arterioarteriellem thrombembolischen distalen ACI- und Mediaverschluss gestellt. Da große Teile des Stromgebiets der A. cerebri media noch nicht infarziert waren, wurde interdisziplinär mit der Neuroradiologie die Indikation zur notfallmäßigen endovaskulären Rekanalisation (Thrombektomie der A. cerebri media und ggf. Stenting der ACI) gestellt.

**Abb. 2:**
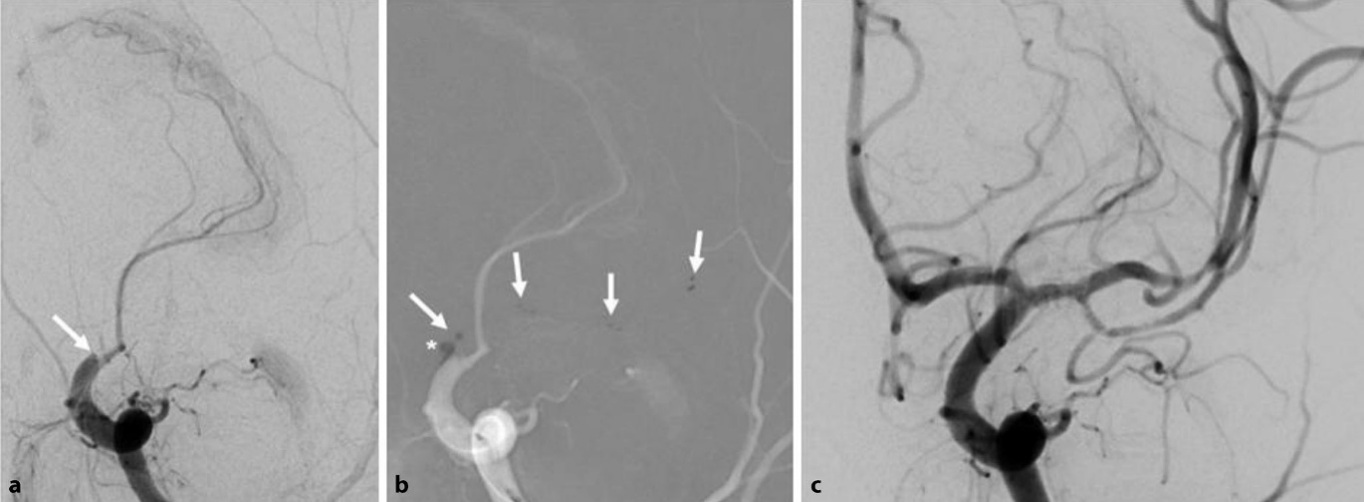
Endovaskuläre Intervention (Thrombektomie). **a** Ausgangsbefund (KM-Injektion in die linke A. carotis interna, p.-a.-Ansicht) mit Abbruch der Arterie distal-intrakraniell, **b** Stent-Retriever mit punktförmigen röntgendichten Markierungen (*Pfeile*) im verschlossenen Gefäß entfaltet, Aspirationskatheter mit röntgendichter Spitze (*Sternchen*) direkt am Thrombusbeginn, **c** Endergebnis mit rekanalisierten Gefäßen

Bei der neuroradiologischen Intervention (Abb. 2) konnte zunächst der Verschluss der distalen ACI und des proximalen Mediahauptstamms mit einem kombinierten Manöver aus Stent-Retriever-Thrombektomie und Kontaktaspiration am Thrombus rekanalisiert werden. Weitere Thrombektomiemanöver waren notwendig, um Thromben aus dem distalen Mediahauptstamm und weiter peripheren Ästen, die u. a. die motorische Zentralregion versorgten, zu entfernen. Sukzessive gelang so die Rekanalisation des Stromgebiets. Die ACI-Dissektion zeigte keine Lumeneinengung, sodass auf ein Stenting verzichtet wurde.

Die MRT-Verlaufskontrolle (Abb. 1d) zeigte einen Infarktdefekt, der nicht über den initialen Infarkt hinausging, sodass das übrige Mediaterritorium durch die Rekanalisation erhalten werden konnte.

Periprozedural wurde durch repetitive Gaben von (Cafedrin/Theodrenalin) ein hochnormaler MAP > 70 mm Hg angestrebt (altersentsprechender Normwert > 65 mm Hg). Zudem wurden im Anschluss an die erfolgreiche interventionelle mechanische Thrombusaspiration 2‑malig 0,5 mg Nimodipin endovaskulär appliziert, um residuelle Vasospasmen zu lösen.

## Intensivmedizinischer Verlauf und Frührehabilitation

Das Mädchen wurde postinterventionell auf die pädiatrische Intensivstation übernommen und dort extubiert. Die Gerinnungsparameter ergaben neben minimal erhöhten D‑Dimeren einen unauffälligen Befund. Zur postinterventionellen Antikoagulation erhielt das Mädchen Acetylsalicylsäure in prophylaktischer Dosierung (3 mg/kgKG und Tag). Im Multiplate war sie als Responder zu werten. Die TRAP ergab einen grenzwertig niedrigen Befund, sodass ASS auf 100 mg einmal tägl. (= 5 mg/kgKG und Tag) p.o. erhöht wurde. Es bestanden zunächst weiterhin eine schlaffe Parese rechts, eine zentrale Fazialisparese sowie eine Aphasie. Zur frühzeitigen Förderung wurde eine physiotherapeutische Beübung begonnen. Hierunter zeigten sich erste aktive Fußbewegungen. Am 4. postinterventionellen Tag wurde das Mädchen auf die pädiatrische Normalstation verlegt.

Hier verbesserte sich unter supportiver Physiotherapie der klinisch-neurologische Zustand im Verlauf. Sie zeigte eine zunehmende Spontanmotorik der rechten oberen und insbesondere auch der rechten unteren Extremität; das Gangbild wurde täglich stabiler. Am rechten Arm konnten erste Greifversuche mit der Hand verzeichnet werden. Sprachlich bildete die Patientin zunehmend 4 Wortsätze, bei uneingeschränktem Sprachverständnis. Sie wurde 13 Tage nach der Intervention nach Hause entlassen. Von hier wurde wenige Tage später eine stationäre Phase-C-Neurorehabilitation angetreten.

In der 3 Monate nach dem Ereignis durchgeführten erweiterten Gerinnungsdiagnostik ergab sich kein sicherer Hinweis auf eine Gerinnungsstörung oder Thrombophilieneigung. Die Thrombozytenaggregation mit ASS wird für insgesamt ein Jahr fortgeführt. In der MRT-Verlaufskontrolle stellten sich ein postischämischer Defekt im Bereich der Stammganglien links sowie eine mäßige links parietal betonte Atrophie sowie Verschmälerung des Crus cerebri links dar. Die A. cerebri media sowie anterior links ist vollständig rekanalisiert.

In der neurologischen Verlaufskontrolle 5 Monate nach dem Ereignis zeigte das Mädchen eine armbetonte spastische Hemiparese rechts. Die Fazialisparese war bis auf eine minimale Mundwinkelasymmetrie regredient.

## Zusammenfassung/Diskussion

Der vorliegende Fall zeigt eindrücklich die potenziell fatalen Folgen eines Bagatelltraumas im Kopf-Hals-Bereich als Auslöser einer traumatischen intrakraniellen Gefäßdissektion mit nachfolgender Ischämie. Diese stellt zwar im Gesamtkollektiv der zerebralen Perfusionsstörungen insgesamt eine seltene Entität dar (so beschrieben Lee at al. in ihrer Studienpopulation eine kombinierte Häufigkeit von 2,6/100.000 über alle Altersgruppen bei intrakraniellen Dissektionen, wobei etwa zwei Drittel der Fälle die A. carotis interna betrafen und sich nur ein Drittel im Bereich der A. vertebralis manifestierte), dennoch kann diese Schlaganfallursache in allen Altersgruppen auftreten und ist insbesondere im Kindes- und Jugendalter für bis zu 25 % aller Schlaganfälle verantwortlich. Zusätzlich kommen extrakranielle Dissektionen mit meist vergleichbaren Auslösern und entsprechender Symptomatik vor. Neben klassischen Auslösern, d. h. vor allem Verkehrsunfällen und Stürzen mit ausgeprägter Flexion und Extension der HWS, sind unzählige scheinbar belanglose Auslöser beschrieben, so z. B. sämtliche sportlichen Aktivitäten, inklusive Tanzen, Yoga und Trampolinspringen, zudem Fahrten in Achterbahnen und anderen Fahrgeschäften sowie sogar Dissektionen nach Husten- oder Niesattacken [1–7].

Nachdem ein relevanter Anteil der Dissektionen zunächst klinisch inapparent bleibt, lässt sich häufig kein konkreter Auslöser benennen, da das auslösende Ereignis nicht mehr präsent ist. Eine genetische Disposition durch angeborene Bindegewebsdefekte wie Marfan- oder Ehlers-Danlos-Syndrome scheint zwar plausibel, lässt sich allerdings anhand der Epidemiologie nicht nachweisen. Allerdings zeigte auch unsere Patientin eine anatomische Auffälligkeit im Sinne einer Aplasie des M. sternocleidomastoideus der betroffenen Seite. Dies könnte einerseits durch eine unzureichende Stabilisierung des Schädels während des Traumas und andererseits durch einen abnormen Verlauf der extrakraniellen Karotisanteile zur entstandenen Schädigung beigetragen haben. Zwar finden sich in der Literatur vereinzelte Fallberichte über unilaterale Fehlanlagen der unteren Halsmuskulatur, insbesondere des M. sternocleidomastoideus und des M. trapezius, ein relevanter Krankheitswert jenseits der entsprechenden Fehlhaltungen (insbesondere Torticollis) ist bislang nicht beschrieben. Auch Hinweise auf ein gehäuftes Auftreten kranio- und zerebrovaskulärer Pathologien finden sich bislang nicht [8–12].

Das Management sollte interdisziplinär in Zentren mit hoher Expertise in der Versorgung von Schlaganfällen erfolgen. Hierfür ist eine SOP für den pädiatrischen Schlaganfall vorzuhalten. Neben den Grundpfeilern der neurologischen Intensivmedizin spielen insbesondere eine mechanische Reperfusionstherapie via Thrombusaspiration, ggf. gefolgt von einem Stenting des betroffenen Blutgefäßes, sowie die systemische oder lokale Lysetherapie führende Rollen. Eine Sekundärprophylaxe mit Thrombozytenaggregationshemmern sollte in der Regel zeitlich begrenzt durchgeführt werden. Die Prognose ist – bedingt durch das jüngere Durchschnittsalter verglichen mit anderen Schlaganfallformen – günstiger. Dennoch ist häufig mit Residuen zu rechnen. Die klinische Untersuchung bei pädiatrischen Patienten ist Compliance-bedingt im Vergleich zum Erwachsenen deutlich erschwert und erfordert eine hohe Expertise. Umso wichtiger sind eine entsprechende Versorgung in einem Zentrum sowie die Würdigung potenzieller klinischer Frühsymptome wie in unserem Fall einer fluktuierenden Hemiparese mit motorischer Aphasie sowie ein zügiges interdisziplinäres Management.
